# An investigation into the pharmaceutical advertising in Iranian medical journals

**DOI:** 10.1186/s40545-022-00415-1

**Published:** 2022-03-07

**Authors:** Nazila Yousefi, Zahra Sharif, Fateme Chahian, Tayebe Mombeini, Farzad Peiravian

**Affiliations:** 1 Department of Pharmacoeconomics and Pharma Management, School of Pharmacy, Shahid Beheshti University of Medical Sciences, Tehran, Iran; 2 School of Pharmacy, Alborz University of Medical Sciences, Karaj, Alborz Iran; 3 Islamic Azad University of Pharmaceutical Science, Tehran, Iran; 4 School of Pharmacy, Shahid Beheshti University of Medical Sciences, Tehran, Iran

**Keywords:** Pharmaceutical advertisements, Medical journals, Regulation, Compliance, Social control

## Abstract

**Background:**

Pharmaceutical advertising is not only considered a key factor in the successful launch of pharmaceutical products, but is also an important source of public health information with a significant impact on consumer choice and behavior. Nowadays, advertising has become the broadest dissemination channel for various products, including medicines, which may ultimately lead to the generalization of self-treatment or mistreatment. Improper drug promotion can exacerbate unhealthy outcomes by making false or misleading claims, using inferior references, and failing to meet international standards. This study aimed to examine the requirements for pharmaceutical advertising from regulatory perspective and the compliance of Iranian pharmaceutical advertisements to related standards and guidelines. It is limited to print advertisements in Iranian national medical journals and magazines.

**Method:**

The present study is a descriptive–analytical study using bibliometric methods. As a first step, a comprehensive review of the national and international regulations on drug advertising was conducted and a comparison of different regulations was provided. In the second step, a checklist was created to evaluate the compliance of drug advertising with the extracted regulations.

**Result:**

The results of the present study show that the claims made in Iranian drug advertisements are 29.10% valid, 27.67% exaggerated, 23.10% controversial, 12.62% misleading, and 6.8% invalid. In general, we found that most medical advertisements in Iranian journals and magazines comply with national laws and regulations. However, many international requirements are not met in these advertisements.

**Conclusions:**

Although we found that printed medical advertisements in Iran are roughly compliant with national regulations, there is still a long way to achieve full compliance. Monitoring processes should be improved and clearly defined penalties should be set to avoid misleading claims and their likely health consequences. It is very important in Iran to update the existing rules and regulations for medical advertisements according to international guidelines. More careful monitoring of the content of advertising and the accuracy of claims are also needed.

## Background

The growing trend towards healthy lifestyles and health consciousness is a global phenomenon. Pharmaceutical industry is one of the most critical industries due to its health and financial aspects [1]. The global pharmaceutical market amounted to $1217 billion in 2019, with a compound annual growth rate (CAGR) of 6.7%. By the end of 2019, the annual value of Iran's pharmaceutical industry had hit €2.5 billion [2].

Medicines can treat acute diseases, improve chronic conditions, relieve symptoms, and support future health. However, a decision must be made about its benefits and potential harms before using a drug. To make an informed decision, one needs information about the goals of the treatment, how it works, how it should be used, the potential benefits and harms, and a comparison of the drug to other available treatments or other products [3].

Investment in pharmaceuticals has recently increased and now accounts for the largest share of healthcare budgets [4]. Part of the increase in drug costs is certainly due to the growing number of effective drugs. However, there is concern that rising costs are often the result of increased advertising of drugs that do not necessarily provide more effective or efficient treatment [5].

Pharmaceutical advertising is not only recognized as a key factor in the successful launch of pharmaceutical products, but is also an important source of public health information with a significant impact on consumer choice and behavior [6].

Today, major pharmaceutical companies cannot succeed without advertising. Considering the fundamental changes in society due to a large amount of information received, the lack of geographical boundaries in advertising, and the easy access to the Internet and social networks, advertising has become the broadest dissemination channel for various products, including medicines, which may ultimately lead to the generalization of self-treatment or mistreatment. According to the World Health Organization (WHO), the number of deaths resulting from improper and unauthorized administration of medicines is the fifth leading cause of death worldwide [7, 8]. Improper drug promotion can exacerbate this unhealthy outcome by making false or misleading claims, using inferior references, and failing to adhere to international standards [9].

Drug promotion can be directed to patients or the community of physicians and healthcare providers. Proponents of direct-to-patient prescribing claim that advertising informs consumers about possible illnesses and potential treatments and encourages people to see a doctor. They believe the information provided this way strengthens the doctor–patient relationship and influences their doctors' decisions. On the other hand, opponents point to safety concerns, rising costs, disruption of doctor–patient communication, and substance abuse [10]. This type of advertising is banned in most countries. The United States and New Zealand are the only two countries where advertising prescription drugs directly to patients is legal.

In one study, Lizuka and Jane examined the impact of direct-to-consumer advertising. Regarding the impact of direct-to-consumer advertising on physicians' selection of antihistamines, they found that it had little effect [11]. Stremersch cites the lack of research on direct-to-consumer advertising, patient inquiries, and patient–supplier relationships. Direct-to-consumer advertising does not directly affect physicians' prescriptions but directly impacts patients' requests [12].

Maine et al. studied drug advertising and its infiltration in the United States. The goal of the Division of Drug Advertising is to determine whether consumers are provided with truthful information and rational reasons regarding health problems, treatment options, and advances in medical science. Their study compared direct-to-consumer advertising (DTC) for prescription drugs and over-the-counter (OTC) drugs and dietary supplements using content analysis. The results showed that direct-to-consumer advertising is not only based on logical prompts, but also uses more positive and negative emotional experiences than over-the-counter drugs or dietary supplements [13].

Meanwhile, there is also criticism of over-the-counter drug advertising. OTC medicines can be obtained at a pharmacy or grocery store for the symptoms of cold, severe pain, and other simple illnesses. Public health organizations have flagged up the widespread abuse of over-the-counter medications because it can lead to the same serious side effects and health problems as the use of illegal drugs [14, 15]

The Food and Drug Administration (FDA) has regulated advertising since 1962 to ensure that it is not misleading.

One of the most important ways to protect consumer health is to regulate the advertising and marketing of pharmaceutical and healthcare products and to promote professional ethics in the trade of these products. Ethical and legal liability can protect consumers' corporeal and spiritual interests and help the profession maintain the market [16].

Peek et al. examined pharmaceutical DTC and OTC advertising in Korea, Japan, Hong Kong, Australia, and the United States in terms of current and future developments. Their study aimed to overview the regulations, studies, and practices of drug advertising in these countries. They concluded that the similarities toward DTC and OTC advertising regulation among these nations outweigh the differences. However, DTC advertising is just allowed in the United Sates and New Zealand. It is banned in other countries [17].

Vlassov et al. investigated whether drug advertisements in Russian medical journals provided the necessary information for a safe prescription. All advertisements published in all sections of selected journals were reviewed in 1998. Almost all of them had withheld the basic information needed for a proper prescription, leading to misunderstandings [18].

The study by Valanova et al. shows that due to the growing movement of evidence-based medicine in physicians' prescribing behavior, the pharmaceutical industry has established links between bibliographic references and clinical trials for prescription drugs. Another study conducted in Spanish medical journals shows that all advertisements for antihypertensive and lipid-lowering drugs published in six Spanish medical journals contained at least one bibliographic reference. However, the conclusion is that physicians should be cautious when evaluating advertisements claiming that a drug is more effective, safer, or more pleasant, even if these claims are accompanied by bibliographic sources and clinical trials published in reputable medical journals [19].

In a study evaluating drug advertisements in Indian and non-Indian medical journals, it was found that 56.66% of advertisements in Indian journals and 86.67% in non-Indian journals had mentioned adjuvants. The involvement of pharmaceutical companies in building a commercial relationship with physicians, compromising the educational aspect of scientific information about medicines, is one explanation for the failure of pharmaceutical companies to meet all the criteria of WHO and Codes of Ethical Drug Advertising [20].

In another study, drug advertising was evaluated using Health Action International (HAI) criteria. This involved formulating detailed criteria for evaluating drug advertising that further develops and extends the WHO criteria. No advertisement met all of the criteria. In addition, the vast majority of drug advertisements were found to lack information on harms and to have limited evidence to support the claims they made [21].

In a cross-sectional study of medical advertising published in the Journal of the Danish Medical Association in 2015, the most commonly advertised drugs had no proven substantial added benefit over older treatments, while being substantially more expensive [22].

Against this background, in this study, we attempt to show the extent to which pharmaceutical advertising in Iranian medical journals complies with regulations after comparing pharmaceutical and healthcare advertising laws in Iran and some other countries. This study is a descriptive cross-sectional study that was conducted in two phases. In the first phase, a literature review was conducted, and a checklist was developed to assess compliance with national laws and international guidelines for pharmaceutical advertising. In the second phase, the extent of this compliance was investigated by evaluating some advertisements taken from medical journals in a year. In this research, after explaining the laws on pharmaceutical and healthcare advertising in Iran and some other countries, an attempt is made to provide evidence of (non-)compliance with national and international laws in Iran.

## Method

This study was a descriptive–analytical study using bibliometric methods. This study examines the requirements for pharmaceutical advertising from the perspective of regulatory agencies. It is limited to print advertisements in national medical journals and magazines and does not include other forms of advertising. As a first step, a comprehensive literature review was conducted to review the national and international regulations governing drug advertising and papers published papers in this era. A checklist was then created to assess the compliance of drug advertising with the extracted regulations.

Due to the diversity of regulations in different countries on drug promotion, we intentionally selected a wide variety of countries, ranging from highly regulated to deregulated countries. Therefore, the regulations of the United States, WHO, China, the United Arab Emirates, Denmark, India, Australia, and Iran were reviewed. Then, a comprehensive checklist was developed to assess compliance with drug advertising regulations in Iranian medical journals.

All drug advertisements published in print or electronic form in journals and magazines from 2019 to 2020 were considered the research population. According to our survey approach to data collection, 124 journals were considered the population, and we contacted all of them to receive the issues published in the said year and obtain their informed consent. Finally, 20 journals were selected (16% response rate), including 256 issues in the study period.

The checklist prepared by the researcher consists of 6 sections, as shown in Table 1.

**Table 1:**
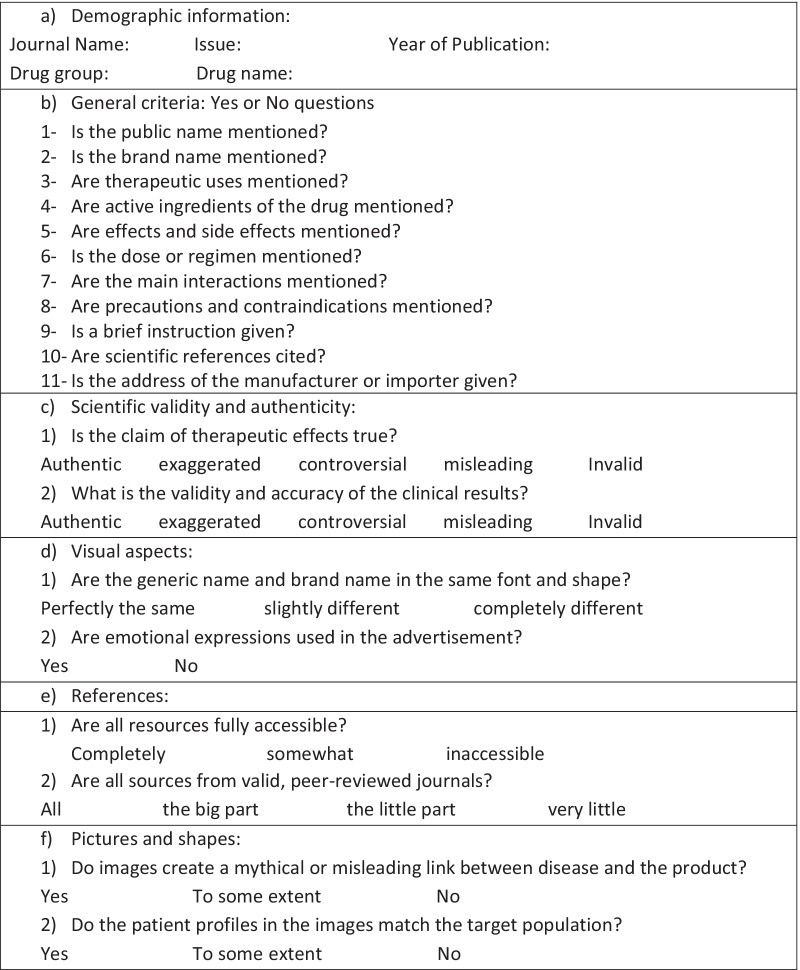
Checklist developed to evaluate drug advertisements in Iranian medical journals

The evaluation team reviewed the drug advertisements published in the selected journals, consisting of a pharmacist, a general practitioner, and a clinical pharmacist, using the checklist. The information was then analyzed using SPSS software 25.

Finally, to weigh and rank the violations, the Delphi method was used with the help of a panel of experts. For this purpose, each drug advertising criterion listed in Table 1 was assessed for importance in 3 rounds. This includes group discussions, repetition, controlled feedback, statistical analysis of responses, and calculation of consistency and convergence of responses[23]. The Delphi was conducted with 6 experts, and a consensus was reached in the second round.

## Results

### Snapshot of the national and international regulations on pharmaceutical advertisements

- *WHO* The *WHO* standards for the basic requirements of drug advertising have become a basic model for drug advertising laws in many countries. In accordance with the ethical standards of the *WHO*, the following information must be included in drug advertising in magazines and medical journals: international standard names (INN) or generic names of approved active ingredients, brand name, quantity of active ingredients in each dosage form, names of other known ingredients with possible side effects, approved therapeutic uses, therapeutic regimens, side effects, especially the main side effects of drugs, precautions, contraindications, warnings, the main interactions with food and drugs, name and address of the manufacturer or license holder, and appropriate citations from scientific sources[24].
- *USA* The *Federal Food and Drug Administration (FDA)* and the *Controlled Drug and Substance Administration (CDSA)* have established a general framework for advertising pharmaceutical products, emphasizing that advertising must not be untrue, deceptive, or misleading as to quality, ingredients, or safety. In addition, all advertising must be based on its marketing authorization [25]. In addition, any type of advertising that directly or indirectly promotes the sale of drugs is prohibited. In the U.S., advertising must be reviewed and approved by regulatory agencies, and any advertising prior to approval is prohibited. In addition, the *FDA* imposes penalties of up to two years in prison or a fine of up to $5 million for failure to comply with advertising regulations [26]. If contract manufacturing is involved, this should be reflected in the advertisement. Other general requirements include the following rules: advertising must be accurate, complete, clearly designed, credible, and trustworthy; must mention the brand name and generic name of the drug; must emphasize the rational use of the drug; and must provide clear information to assess risk. While maintaining privacy, Internet advertising must meet all these standards. Since advertising is restricted to the general public, it must take necessary measures to prevent websites intended for healthcare professionals from accessing it [26].
- *Australia* Pharmaceutical advertising in *Australia* must include the product brand, active ingredient names approved in *Australia*, supplier name and address, product information form (with information such as approved signs, contraindications, important clinical warnings, precautions for use, and side effects), and a statement that physicians must review the full product information before prescribing. Advertising over-the-counter drugs to the public is legal. But all related advertising published in newspapers, magazines, films, on billboards, on posters, on radio, and on television must be approved in advance [17].
- *Denmark* In *Denmark*, pharmaceutical advertising is defined as any dissemination of information, activities to promote the prescription, supply, and sale of pharmaceutical products, and all should follow ethical principles [27].
- *China* The Chinese standards for pharmaceutical advertising state that certain information must be included in pharmaceutical advertising, regardless of whether the advertising is directed to the general public or healthcare professionals [28]. This information includes the advertising license number, the drug manufacturing license number, the common name of the drug if it is a prescription product, the phrase "For pharmacists and pharmaceutical professionals only", and the phrase "Buy and use according to the instructions or under the guidance of a pharmacist". And if it is a non-prescription product, the advertisement must also include the name of the manufacturer or trading company of the drug and an OTC logo.

The advertising of all pharmaceutical products must be pre-approved by the government. In addition, it is prohibited to compare pharmaceutical products with other drugs in terms of efficacy and safety, even if there is evidence to support it. Defamation of other products is also prohibited. Pharmaceutical companies should not advertise unregistered or unlabeled products. It is forbidden to give information in advertising that scares professionals. According to the standards for drug advertising, advertising for prescription drugs is printable only in medical or pharmaceutical journals jointly determined by the Ministry of Health and the Food and Drug Administration and may not be advertised to the public. Advertising of OTC drugs to the general public, on the other hand, is permitted. Press releases about a new product without mentioning the name of a specific pharmaceutical product or its manufacturer are permitted.

-*United Arab Emirates* Pharmaceutical advertising in the *United Arab Emirates* may be in written, photographic, printed, poster, or sales form or may come as product packaging of any type of media. Regardless of the form, the government must approve it prior to publication and must not violate morality, decency, *UAE* customs, or Islamic values and traditions. In addition, it must not be inaccurate, misleading, or distorting, nor should it prevent potential patients from being informed about the advertised drug's efficacy or potential side effects. Comparative advertising with another product is not permitted. The use of healthcare professionals for medical or drug advertising and advertising directed at children are not allowed [29].
  - *India* In *India*, pharmaceutical advertising includes any notice, circular, label, package, or other documents, as well as any notice produced or transmitted orally or by light, sound, or smoke. Since the purpose of promotional materials is to provide healthcare professionals with sufficient information for prescribing or use, at a minimum, the following information must be clearly and legibly stated and incorporated: the license number for the product, the name and address of the licensee or brand, the address of the part of the company responsible for selling medicinal products on the market, the list of active ingredients, the recommended dose, the method of use, the route of administration, possible adverse reactions, warnings and precautions, and the date the information was prepared or last updated. A pharmaceutical company's merchandise must not influence healthcare professionals, consider financial incentives, and use their name or photograph in promotional materials. The comparison of pharmaceutical products with other medicines must be real, fair, and demonstrable, and requires the prior consent of the beneficiary companies. There are no specific restrictions on advertising over-the-counter medicines if the general standards are met [30].
  - *Iran* Pharmaceutical advertising in *Iran* must not contradict the logical, scientific, and ethical criteria for prescribing medicines or override the independence of the physician's opinion. The payment or offer of financial or non-financial support for the prescription, recommendation, purchase, and consumption of products is prohibited. Information must not be concealed and should always be based on scientifically sound documentation. The results must be fully and transparently mentioned when the drug is introduced by citing scientific sources after obtaining legal approvals. The introduction and marketing of medicines are limited to the medicines in the "List of Iranian Medicines". Direct introduction of medicines to the public, including through mass media, is prohibited. The introduction of medicines is allowed only by pharmaceutical companies licensed by the *Ministry of Health and Medical Education (MoHME)* or an organization of the medical system. It is allowed to announce legally acquired licenses from the relevant official drug authorities. Nevertheless, the propagandistic use of the logo of the Ministry of Health and Medical Education and the *Iran Food and Drug Administration (IFDA)* in the launch is prohibited. Pharmaceutical companies manufacturing or importing drugs are not allowed to introduce their drugs until they obtain a manufacturing or import license from the *IFDA*. Full details of the pharmaceutical company and the manufacturer or importer, including name, address, telephone number, and email address, must be provided in all matters relating to the introduction of medicines. If the drug license specifies special conditions for the prescription or consumption of drugs, this must be mentioned in the introduction. Any activities to set prices to encourage prescription or purchase of medicines are prohibited. Drug advertising in the media is allowed only in medical journals and on websites specifically aimed at the medical audience, or this type of activity is mentioned in the license. Introduction of the drug through the presentation of drug samples, brochures, and catalogs, participation in conferences and personal meetings, lectures, sending information by mail or email is allowed only for the medical audience in compliance with the rules. Information about the stock of a particular drug, mentioning only the name of the drug and the place of delivery of the drug, is allowed only after obtaining permission from the *IFDA*.

Advertising is defined by the Ministry of Culture and Islamic Guidance as the publication of any type of advertisement through public and private media, audio and video channels such as radio, television, satellite networks, press, cinema, Internet, intranets, corporate audio-visual networks, billboards, exhibitions, and printed materials such as catalog sheets, brochures, manuals, packaging, stickers, business cards, slides, banking information, and the like to introduce materials, goods, and services covered by this regulation. Advertising is allowed only for the relevant persons or institutions that have obtained a license from the *MoHME* and other relevant authorities to establish, collect, manufacture, import, and distribute the product. Advertising of medicines and therapeutic properties approved by the *MoHME* is allowed only for holders of medical professions and related institutions. Advertising of medicines is prohibited, as is the mention of curative properties in advertising food, beverages, and cosmetics in public media. If special conditions are specified in the manufacturing or import license for these materials, this must be mentioned in the advertising.

Table 2 summarizes the main components of advertising regulations in some countries.

**Table 2:** Comparison of the most important rules for pharmaceutical advertising in the selected countries

Constitutions	Iran	Australia	India	Denmark	China	FDA/USA
Necessity of inserting a specific drug name	*	*	*	*	*	*
Necessity of inserting a generic drug name	*	*	*	*	*	*
The need to obtain a license or permit to advertise drugs	*	*	*		*	*
Prohibition of direct advertising to consumers	*					*
Need to include the name of the drug manufacturer or importer	*	*		*		*
Prohibition of advertising drugs in popular magazines and periodicals	*				*	
Prohibition of the use of the logos of regulatory authorities	*					
Prohibition of financial or non-financial rewards or incentives	*		*			
Prohibition of plagiarism	*			*		
Prohibition of providing misleading information					*	*
Requirement of transparency and reliability of information		*		*		*
Need to mention precautions and contraindications		*	*	*		*
Need to mention side effects		*	*	*		*
Inclusion of a list of active ingredients		*	*	*		*
Prohibition of comparative advertising			*		*	
Prohibition of advertising for an unregistered product	*				*	
Respect for values and traditions						
Prohibition of targeting children					*	
Prohibition of direct or indirect undermining of competing products					*	

### Empirical study of regulatory compliance

We reviewed the criteria of *WHO* regarding pharmaceutical advertising in 20 journals and 256 ads (Table 3). Guideline compliance was investigated in three different groups of pharmaceutical products: medicines, supplements and herbals. Product brand name rated as the most adapted criteria by Iranian medical journals and magazines in all groups of products. Almost all herbal and supplements, and most of medicines advertisements had instruction to use. However, main contraindications, precautions and providing the scientific references were among the most frequently ignored criteria in all groups of products. Advertisements of herbal medicines completely lacked scientific references and precautions; whereas, 14% of medicines advertisements provided scientific references and 29% of supplement advertisements warned precautions. Only 3% of advertisements on medicines minded side effects, however, near half of herbal advertisements announced about side effects.

**Table 3:** Compliance with WHO criteria for pharmaceutical advertising among Iranian medical journals (total count = 256)

Criteria	Medicines (*N* = 107)	Supplements (*N* = 98)	Herbal drugs (*N* = 51)
Generic name	74%	50%	0%
Brand name	100%	97%	100%
Indications	48%	45%	50%
Active ingredients	2%	23%	48%
Side effects	3%	0%	48%
Dose or diet	19%	12%	37%
Main contraindications	1%	0%	1%
Use precautions	0%	29%	0%
Instruction to use	64%	90%	100%
Scientific references	14%	5%	0%
Importer’s or manufacturer’s address	69%	65%	92%

Then, the status of validity and scientific authenticity of advertisements content were evaluated. The experts' answers to the two main questions listed in Table 4 show the level of the criteria authenticity, exaggeration, controversy, misleading, and invalid information. These criteria were defined to the experts as follows:

Authentic: When the claim made in the ad is agreed upon by the majority of researchers and used in practice, and its indication and clinical results are confirmed.
Exaggerated: The claim made in the ad is agreed upon by the majority of scholars, but in practice it is used sparingly and its clinical results are not confirmed.
Controversial: The claim made in the ad has been agreed upon by a handful of scholars, but in practice it has not been used and there are doubts about its clinical results.
Misleading: The claim made in the ad was not agreed upon by the majority of scholars in practice, was not used, and clinical results not confirmed.
Invalid: When no credible research has been done on the claim made in the ad.

**Table 4:** Compliance in terms of validity and scientific authenticity (total count = 256)

	Authentic (%)	Exaggerated (%)	Controversial (%)	Misleading (%)	Invalid (%)
The accuracy of the claim about the therapeutic effect	26	25	20	11	5
The accuracy of the scientific clinical results	33	29	25	14	7

After that, a visual evaluation of the misleading images was performed using the four main criteria listed in Table 5.

**Table 5:** Status of text and form (total count = 256)

Percentage of compliance	Perfectly the same	Slightly different	Completely different
The same generic name and brand font and shape	21%	54%	25%

	Yes (%)	To some extent (%)	No (%)
Demonstrating a mythical or misleading link between a disease and a product	57	27	16
If the patient profiles match the target population	7	8	85

	Yes	No
Presence of emotional expressions in advertising	32%	68%

Finally, the accessibility of the references and their validity were evaluated, as shown in Table 6. The experts were asked to rank the validity of references as follows:

The big part: If 50% and more of the references were valid.
The little part: If less than 50% to 20% were valid.
Very little: If less than 20 percents of references were valid.

**Table 6:** Valid references (total count = 256)

Percentage of compliance	Completely	Somewhat	Unrecoverable
Accessibility	11%	8%	79%

	All	The big part	The little part	Very little
Validity	2%	3%	41%	54%

## Discussion

There are various laws and regulations governing drug advertising at the regional, national, and state levels. In addition, some countries have general trade laws for businesses that also affect drug advertising. For example, anti-bribery and anti-corruption laws have a significant impact on pharmaceutical companies' interactions with healthcare professionals worldwide. The purpose of these laws and regulations is to prevent improper pharmaceutical marketing activities through executive action.

Drug promotion rules and regulations fall into three general categories: (I) rules and regulations on the basic requirements for pharmaceutical advertising, such as the essential information that must be included in advertising; (II) rules and regulations on product claims, including the effectiveness of the product; and (III) rules and regulations on the interaction and communication of pharmaceutical companies with healthcare professionals and caregivers [31].

Although many countries have laws and regulations on the information that must be included in pharmaceutical advertising, the quality and extent of the information vary.

This study shows that in Iran, the trade name of the drug (99.53%), generic name (64.19%), order of consumption (71.96%), and address of the manufacturer or importer (71.03%) are among the disclosures that account for the highest percentage in domestic drug advertisements. Next come the indication of the drug (46.73%), the regimen (43.23%), scientific references (11.21%), side effects (3.10%), drug interaction (0.83%), and precautions and contraindications (0.47%). In other words, side effects, drug interactions, precautions, and contraindications are found in less than 5% of domestic drug advertisements.

In practice, these elements are often lost even in drug advertising in industrialized countries. A systematic review of the quality of drug advertising in medical journals examined 24 studies of advertising in 26 countries. The results of this meta-analysis indicated that while most advertisements included the brand name and generic name of the product, other information needed for rational prescribing, such as contraindications, interactions, side effects, warnings, and precautions, was usually missing [32]. It is important to note that this important information is required by the International Federation of Pharmaceutical Manufacturers & Associations (IFPMA) and the European Federation of Pharmaceutical Industries and Associations (EFPIA) [33]. A 2001 study in Russia showed that only 45% of drug advertisements met the criteria and laws for drug advertising [18]. Results for the United Republic of Tanzania (40%) and Italy (34%) also show little compliance [34].

Similarly, in the USA (2008), nearly half of the drug advertisements published in U.S. medical journals did not adhere to at least one of the FDA regulations. In addition, drug advertisements do not adequately convey the initial information needed by physicians for safe prescribing. Most do not mention serious risks, and almost half of them do not cite verifiable scientific references [26]. Less than 50% of advertisements in Zimbabwe include information about side effects, warnings, precautions, or interactions with other medications [35]. Similarly, in Nepal, two-thirds of advertisements mentioned the side effects of drugs for physicians, as did precautions, contraindications, or warnings [36]. Similarly, only half of most of the 200 cases of drug advertising in 2014 met the criteria of the WHO for rational drug advertising [37].

In addition, the claims in any advertisement must be true and not misleading. This means that medical facts must not be omitted or half-heartedly stated. The claims must provide balanced scientific evidence, and they simply must not provide half the picture. The present study showed that the claims made in Iranian drug advertisements were 29.10% accurate, 27.67% exaggerated, 23.10% controversial, 12.62% misleading, and 6.8% invalid.

A comparative study of advertisements in medical journals in Australia, Malaysia, and the United States showed that most of the claims were vague, low quality, and less than one-third of them were considered authentic [38]. In Germany, in 2004, 94% of claims in physician brochures were not supported by scientific evidence, and no scientific reference had been mentioned in 15% [39]. The situation in low- and middle-income countries is even more worrying than in high-income countries. A 2006 study in Bangladesh found that 34% of the information in 116 brochures for family physicians was misleading [40].

## Conclusion

This study showed that most medical advertisements in Iranian journals and magazines comply with national laws and regulations. This means that it is imperative in Iran to update the existing rules and regulations for pharmaceutical advertising according to international guidelines.

Although we found that the compliance of printed drug advertising in Iran with national regulations is approximately high, there is still a long way to achieve full compliance. Therefore, monitoring processes should be improved to avoid misleading claims and their likely health consequences, and clear sanctions should be imposed for non-compliance. More careful monitoring of the content of advertising and the accuracy of claims is also needed.

## Data Availability

The dataset(s) supporting the conclusions of this article is(are) included within the article (and its additional file(s)).

## Abbreviations

CAGR: Compound annual growth rate
WHO: World Health Organization
DTC: Direct-to-consumer advertising
OTC: Over-the-counter
FDA: Food and Drug Administration
HAI: Health Action International
INN: International Standard Names
CDSA: Controlled Drug and Substance Administration
MoHME: Ministry of Health and Medical Education
IFDA: Iran Food and Drug Administration
IFPMA: International Federation of Pharmaceutical Manufacturers and Associations
EFPIA: European Federation of Pharmaceutical Industries and Associations

## References

[1] Lee B, Salmon CT, Paek H-J (2007). The effects of information sources on consumer reactions to direct-to-consumer (DTC) prescription drug advertising: a consumer socialization approach. J Advert.

[2] HELPDESK. Pharmaceutical Industry In Iran [Internet]. https://sanctions-helpdesk.eu/sites/default/files/2021-04/2020.10.ThePharmaceuticalIndustryinIran_0.pdf (2020) accessed 9 Oct 2021.

[3] Alves TL, Lexchin J, Mintzes B (2019). Medicines information and the regulation of the promotion of pharmaceuticals. Sci Eng Ethics.

[4] Heffler S, Levit K, Smith S, Smith C, Cowan C, Lazenby H (2001). Health spending growth up in 1999; faster growth expected in the future. Health Aff.

[5] Wilkes MS, Bell RA, Kravitz RL (2000). Direct-to-consumer prescription drug advertising: trends, impact, and implications: aiming drug ads at consumers means big business for drug companies, but its effect on clinical care is not yet known. Health Aff.

[6] Lawton VL (2021). Drug discovery and development E-book: technology in transition.

[7] Ventola CL (2011). Direct-to-consumer pharmaceutical advertising: therapeutic or toxic?. Pharm Ther.

[8] Golichenko M, Merkinaite S, Canadian HIV, Network AL (2011). Ukrainian Drugs legislation and the European Convention for the protection of human rights and fundamental freedoms. Can HIV/AIDS.

[9] Spurling GK, Mansfield PR, Montgomery BD, Lexchin J, Doust J, Othman N (2010). Information from pharmaceutical companies and the quality, quantity, and cost of physicians’ prescribing: a systematic review. PLoS Med.

[10] Beltramini RF (2010). DTC advertising’s programmatic research and its effect on health communication. Health Commun.

[11] Iizuka T, Jin GZ (2007). Direct to consumer advertising and prescription choice. J Ind Econ.

[12] Stremersch S (2008). Health and marketing: the emergence of a new field of research. Int J Res Mark.

[13] Main KJ, Argo JJ, Huhmann BA (2004). Pharmaceutical advertising in the USA: information or influence?. Int J Advert.

[14] Sansgiry S, Sharp WT, Sansgiry SS (1999). Accuracy of information on printed over-the-counter drug advertisements. Health Mark Q.

[15] Foxhall K. FDA may restrict acetaminophen. WebMD Heal News Retrieved Sept. 2009;3.

[16] Hosseini M (2009). Consumer rights in advertising and marketing of pharmaceuticals and health products. Iran J Med Ethics Hist.

[17] Paek H-J, Lee H, Praet CLC, Chan K, Chien PM, Huh J (2011). Pharmaceutical advertising in Korea, Japan, Hong Kong, Australia, and the US: current conditions and future directions. Health Commun Res.

[18] Vlassov V, Mansfield P, Lexchin J, Vlassova A (2001). Do drug advertisements in Russian medical journals provide essential information for safe prescribing?. West J Med.

[19] Villanueva P, Peiró S, Librero J, Pereiró I (2003). Accuracy of pharmaceutical advertisements in medical journals. Lancet.

[20] Dabhade SA, Dabhade SS (2021). Evaluation and comparison of drug advertisements published in medical journals using WHO criteria for ethical medicinal drug promotion and OPPI criteria for drug advertisements. Int J Cur Res Rev.

[21] Jha N, Sapkota Y, Shankar PR (2020). Critical evaluation of drug advertisements in a Medical College in Lalitpur, Nepal. J Multidiscip Healthc.

[22] Boesen K, Simonsen AL, Jørgensen KJ, Gøtzsche PC (2021). Cross-sectional study of medical advertisements in a national general medical journal: evidence, cost, and safe use of advertised versus comparative drugs. Res Integr Peer Rev.

[23] Sam Scheele D (1975). The delphi method.

[24] Schenker Y, Arnold RM, London AJ (2014). The ethics of advertising for health care services. Am J Bioeth.

[25] Meredith PA, Elliott HL (1994). FDA guidelines on trough: peak ratios in the evaluation of antihypertensive agents United States Food and Drug Administration. J Cardiovasc Pharmacol.

[26] Korenstein D, Keyhani S, Mendelson A, Ross JS (2011). Adherence of pharmaceutical advertisements in medical journals to FDA guidelines and content for safe prescribing. PLoS ONE.

[27] Greenwood J, Ronit K (1991). Pharmaceutical regulation in Denmark and the UK: reformulating interest representation to the transnational level. Eur J Polit Res.

[28] Chan KKW (1996). Illegal pharmaceutical advertising in China. Gaz (Leiden, Netherlands)..

[29] Gharibyar H, Sharif Y (2012). Evaluation of pharmaceutical drug information brochures in the Emirate of Abu Dhabi (United Arab Emirates). J Pharm Health Serv Res.

[30] Handa M, Vohra A, Srivastava V (2013). Perception of physicians towards pharmaceutical promotion in India. J Med Mark.

[31] Santiago MG, Bucher HC, Nordmann AJ (2008). Accuracy of drug advertisements in medical journals under new law regulating the marketing of pharmaceutical products in Switzerland. BMC Med Inform Decis Mak.

[32] Othman N, Vitry A, Roughead EE (2009). Quality of pharmaceutical advertisements in medical journals: a systematic review. PLoS ONE.

[33] Powrie-Smith A (2017). European federation of pharmaceutical industries and associations. Impact.

[34] Herxheimer A, Lundborg CS, Westerholm B (1993). Advertisements for medicines in leading medical journals in 18 countries: a 12-month survey of information content and standards. Int J Health Serv.

[35] Sibanda N, Gavaza P, Maponga CC, Mugore L (2004). Pharmaceutical manufacturers’ compliance with drug advertisement regulations in Zimbabwe. Am J Health Pharm.

[36] Alam K, Shah AK, Ojha P, Palaian S, Shankar PR (2009). Evaluation of drug promotional materials in a hospital setting in Nepal. South Med Rev..

[37] Ganashree P, Bhuvana K, Sarala N (2016). Critical review of drug promotional literature using the World Health Organization guidelines. J Res Pharm Pract..

[38] Othman N, Vitry AI, Roughead EE (2010). Quality of claims, references and the presentation of risk results in medical journal advertising: a comparative study in Australia, Malaysia and the United States. BMC Public Health.

[39] Tuffs A (2004). Only 6% of drug advertising material is supported by evidence. BMJ.

[40] Islam MS, Farah SS (2007). Misleading promotion of drugs in Bangladesh: evidence from drug promotional brochures distributed to general practitioners by the pharmaceutical companies. J Public Health (Bangkok).

